# Modeling pulmonary fibrosis through bleomycin delivered by osmotic minipump: a new histomorphometric method of evaluation

**DOI:** 10.1152/ajplung.00311.2019

**Published:** 2019-12-18

**Authors:** Francesca Ravanetti, Luisa Ragionieri, Roberta Ciccimarra, Francesca Ruscitti, Daniela Pompilio, Ferdinando Gazza, Gino Villetti, Antonio Cacchioli, Fabio F. Stellari

**Affiliations:** ^1^Department of Veterinary Science, University of Parma, Parma, Italy; ^2^Corporate Preclinical R&D, Chiesi Farmaceutici S.p.A., Parma, Italy

**Keywords:** bleomycin, histomorphometry, lung and skin fibrosis, mouse model, osmotic pumps

## Abstract

The systemic delivery of bleomycin (BLM) to mice through subcutaneously implanted osmotic minipumps may be used to experimentally mimic the typical features of systemic sclerosis and related interstitial lung diseases. The published studies on this model principally have focused on induced dermal modifications, probably because lung lesions are typically mild, subpleurally localized, and difficult to analyze. The use of high BLM doses to increase their severity has been proposed but is ethically questionable because of the compromising of animal welfare. We propose a tailored histomorphometric method suitable to detect and quantify this type of mild lung lesions. Using a two-step automated image analysis, a peripheral region of interest with a depth of 250 µm from the pleural edge was defined on whole slide images, and the fibrotic foci were histomorphometrically characterized. The effects of different BLM doses on lung alterations were evaluated in C57BL/6 mice and 60 U/kg resulted in a fair compromise between fibrotic lesions and animal welfare. This dose was also tested in time course experiments. The analysis revealed a peak of histological fibrotic-like alterations, cytokine expression, metalloprotease, and macrophagic activation between the 21st and 28th day after pump implant. The induced dermal fibrosis was characterized by the progressive loss of the white dermal adipose layer, an increase in dermal thickness, dermal hyperplasia, and more compacted collagen fibers. Despite the trend toward spontaneous resolution, our model allowed a double organ readout of the BLM effect and the identification of a therapeutic window for testing pharmacological compounds without using life-threatening doses.

## INTRODUCTION

Systemic sclerosis or scleroderma (SSc) is a systemic inflammatory disorder of uncertain origin characterized by vascular damage, autoimmunity, and fibrosis of the skin, joints, and internal organs. One of the most widely reported complications arising in scleroderma patients, frequently leading to death, is related interstitial lung disease (SSc-ILD) (26). ILD consists of a group of chronic and progressive disorders in which the pulmonary parenchyma can show varying degrees of fibrosis and inflammation with consequent gas exchange impairment. When inflammation prevails, patients often respond to treatment. Later, a predominance of fibrosis indicates an advanced stage of the disease, which becomes refractory to treatments, rendering patients with poor prognoses (3, 7).

The unclear etiopathogenesis of SSc-ILD and lack of effective treatments require further research. In particular, it is imperative to find highly relevant animal models that are able to reproduce the chronic and progressive aspect of the disease. Lung fibrosis, in fact, spontaneously develops in domestic animals (cats, dogs, etc.) but can provide only limited information (27) toward understanding the pathogenesis and identifying novel therapeutic targets to assess and validate in clinical trials. The most widely used animal model of experimentally induced fibrosis is murine using bleomycin (BLM), a glycosylated peptide antibiotic, isolated from the bacterium *Streptomyces verticillus*. BLM is used in cancer chemotherapy, but it causes adverse side effects, including fibrosis in organs like lungs, in which there is a scarce presence of BLM hydrolase, the deactivating enzyme (11, 13).

Dosage, repetition, and route of administration of BLM may vary in the different murine models (6, 9, 17, 15, 22), producing differences in disease symptoms and histopathological features of the affected organs. Therefore, in performing translational studies to validate novel treatments, it is critical to accurately determine the histomorphometric features of the induced lesions and possibly reproduce the ones typical of the human disease.

In particular, the continuous administration of BLM to mice via subcutaneously implanted osmotic minipumps causes fibrosis of the skin, lungs, and other internal organs (6, 12, 28), reflecting the systemic nature of the human disease; therefore, it is the preferred method used to model human SSc over BLM subcutaneous injections repeated daily for 4–6 wk (28), which require multiple treatments, often cause only localized and self-resolving cutaneous effects, and rarely induce lung fibrosis (13). The pump model, instead, with a single procedure, allows a better standardization of BLM delivery and makes it possible to simultaneously observe the effects of potential antifibrotic therapeutic treatments in different target organs. The pulmonary lesions induced by BLM released by osmotic minipumps have been considered only in a few studies (6, 12, 13, 28), which proved their similarity to those observed in ILDs. The controlled release of BLM produces uniform fibrosis limited to the subpleural portion of the lung, mild inflammation, and presence of large numbers of hyperplastic type II alveolar epithelial cells.

The mildness of the lesions makes it difficult to highlight significant differences among treatments or during the disease time course with a classic histological approach. However, the uniform and predictable pattern of induced fibrosis demonstrated in the literature (6, 12) is promising for better planned experiments, with a more precise estimated number of animals being used in treatment groups and greater reliability of data. Therefore, we believe that the murine osmotic minipump model has strong potential and deserves special attention.

The present study was undertaken to optimize the dosage of BLM administered by continuous infusion via osmotic minipumps to establish in the same animal both dermal and pulmonary fibrosis, mimicking human SSc with ILD-like manifestations. Focusing in particular on the anatomic site in which fibrotic lesions are mainly located after systemic BLM exposure, we developed a tailored histomorphometric method of analysis to better detect, characterize, and quantify mild subpleural fibrosis. First, we subcutaneously implanted osmotic minipumps delivering different doses of BLM to C57BL/6 mice, a strain particularly susceptible to induction of lung fibrosis, to identify the best concentration to obtain a fair compromise between fibrosis and animal welfare. Afterward, we investigated time-dependent changes induced by this dosage in lung morphology, performing an analysis of histological and histomorphometric hallmarks, inflammatory response, and ECM remodeling biomarkers. As other target organs, the skin, liver, and kidney were also considered.

## MATERIALS AND METHODS

### 

#### Experimental animals.

Female inbred C57BL/6 (7- to 8-wk old) mice were purchased from Envigo (San Pietro al Natisone, Udine, Italy). Prior to use, animals were acclimatized for at least 7 days to the local vivarium conditions (room temperature: 20– 24°C; relative humidity: 40–70%; 12:12-h light-dark cycle), having free access to standard rodent food and softened tap water. All animal experiments described herein were approved by the intramural animal welfare committee for animal experimentation of Chiesi Farmaceutici (protocol no. 449/2016-PR) to comply with the European Directive 2010/63 UE, Italian D.Lgs 26/2014 and the revised “Guide for the Care and Use of Laboratory Animals” (19).

#### Bleomycin administration.

**Fig. 1.**
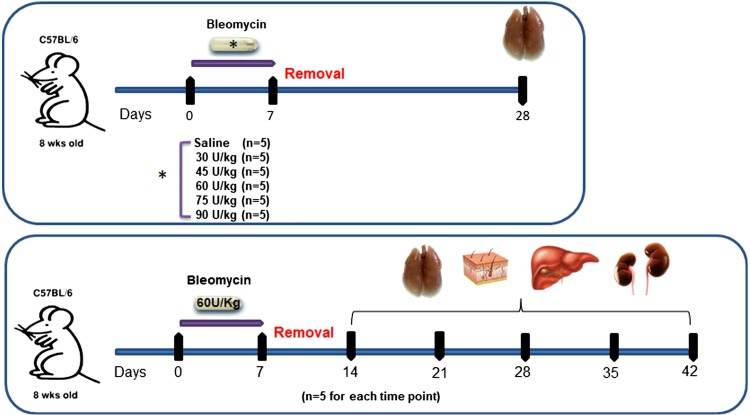
Summary of the experimental model (scale-up and time course). Eight-week-old female C57BL/6 mice were subcutaneously implanted with Alzet osmotic minipumps containing either a 200 µL saline vehicle or bleomycin at different doses. Pumps, implanted under the back skin of mice slightly caudal to the scapulae, continuously delivered their contents at 0.5 µL/h for 7 days and were removed on *day 8*. The dose effect was studied on *day 28*. Each concentration was tested on 5 animals. The time course was performed after 7, 14, 21, 28, 35, and 42 days using the dose of 60 U/kg. For each time point, 5 control animals and 5 experimental animals were used.

Animals were lightly anesthetized with 2.5% isoflurane delivered in a box. BLM (Baxter Oncology GmbH) was administrated to 8-wk-old female C57BL/6 mice using osmotic minipumps (ALZET1007D; DURECT Corporation, Cupertino, CA) containing either 200 μL of saline as vehicle or BLM for 7 days. BLM was dissolved in 200 µL of saline at concentrations of 30, 45, 60, 75, and 90 U/kg. The osmotic minipumps were designed to deliver their contents at 0.5 µL/h for 7 days. On *day 0*, osmotic minipumps were implanted under the back skin of the mice slightly caudal to the scapulae under isoflurane anesthesia. Mice were shaved over the implantation site. A small skin incision was made using surgical scissors, and a subcutaneous pocket was created using the jaws of the hemostat to position the pump. The skin wound was cleaned with Betadine and closed with two surgical clips. Mice were monitored in cages until they reached full recovery. Empty pumps were removed on *day 8*. All mice were weighed daily from the beginning of the trial. The effect of different dosages of BLM was evaluated at the reference point of *day 28* (13). The time course of the selected (60 U/kg) BLM dose effects was assessed at 7, 14, 21, 28, 35, and 42 days after the implantation of the minipumps. After euthanization, at each time point, bronchoalveolar lavage fluid (BALF), lungs, and skin, liver, and kidney samples were collected. Each experiment was performed using five mice per group. The experimental setup is summarized in Fig. 1.

#### Bronchoalveolar lavage, cytokines, and matrix metalloproteinases.

At the selected time points, animals were euthanized with an anesthetic overdose. After euthanization, BALF was collected, as previously described (23, 24), from the bronchial tree by gently washing the airways with 0.6 mL of sterile solution (Hanks’ balanced salt solution was made with a 10× stock and distilled water and supplemented with EDTA to a final 1 mM and HEPES to a final 100 mM) three times. After centrifugation at 400 *g* for 10 min, BALF supernatants were frozen at −80°C for subsequent simultaneous quantitation of multiple cytokines/chemokines using a Bio-Plex Cytokine Assay Kit (Bio-Rad Laboratories, Segrate, Milano, Italy). The cell pellet was resuspended in 0.2 mL of PBS. The cell number was counted with an automated cell counter (Dasit XT 1800J). Murine single-cell suspensions from BALF were resuspended in a FACS buffer (PBS; 0.5% BSA), and red blood cells were lysed using a lysis buffer (BD Bioscience). Cells were then stained using anti-mouse conjugated monoclonal antibodies specific for F4/80 (BioLegend), CD11b (BD Pharmigen), MHC II (BD Pharmigen), and CD206 (Bio-Rad) following datasheet instruction. To first negatively gate out lymphocytes and debris, forward (FSC) and side scatter (SSC) parameters were used together with an F4/80 marker to also negatively gate the neutrophil population (Supplemental Fig. S1; Supplemental Material for this article is available at https://doi.org/10.6084/m9.figshare.11212382). Therefore, we positively selected an F4/80+CD11b+ population, likely representing macrophage cells. In this gate, at each time point observation, the percentage of cells expressing MHC II or CD206 markers was evaluated, representing M1-like and M2-like polarized macrophages, respectively. Data were acquired using a FACS Canto II (BD Biosciences), which collected 20,000 events on the gated F4/80+CD11b+ cells and then analyzed with FACS Diva software. Because we know that using low numbers of surface markers resulted in a poor discrimination of macrophages subsets, we ran in parallel an experiment using bone marrow macrophage-derived cells stimulated with IL-4 and transforming growth factor-β (TGF-β) for 48 h to induce the M2 phenotype and to confirm the accurate expression of the CD206 marker. Bone marrow was flushed from femurs and tibia of C57BL/6 healthy mice and cultured in RPMI + 10%FBS + P/S medium for 7 days with mouse macrophage colony stimulating factor recombinant (Sigma) at 20 ng/mL to have mature macrophages M0. To induce M2 polarization, cells were treated with mouse recombinant IL-4 (20 ng/mL; Sigma) and mouse recombinant TGFβ (20 ng/mL; eBiosciences) for 48 h and subsequently analyzed by FACS.

The concentration of matrix metalloproteinase (MMP) proteins in BALF was determined by enzyme-linked immunosorbent assay (ELISA). MMP-2, MMP-9, and their tissue inhibitor TIMP-1 were measured using the specific ELISA kit (R&D Systems), following the manufacturer’s instructions, as previously described (25).

#### Histology, histomorphometry, and immunofluorescence.

After the euthanization, the lungs, skin, liver, and kidneys were harvested. Lungs were removed and inflated with a cannula through the trachea by gentle infusion of 0.6 mL of 10% neutral-buffered formalin and fixed for 24 h. Skin was harvested on the gluteal region to avoid dermal fibrosis artifact due to proximity to the site of implant. For histological assessment, the samples were dehydrated in a graded ethanol series, clarified in xylene, and paraffin embedded. Sections of 5 μm thickness were cut with a rotary microtome (Slee Cut 6062; Slee Medical, Mainz, Germany). The sections were stained with hematoxylin and eosin (H & E) and Masson’s trichrome (TM) according to the manufacturer’s specifications (Histo-Line Laboratories). The whole slide images (WSI) were acquired by the NanoZoomer S-60 Digital slide scanner (Hamamatsu, Japan) for analysis. Two independent researchers with experience in animal models of lung fibrosis performed blind histological analyses of the specimens/slides. Fibrotic modifications were assessed morphologically and semiquantitatively graded according to the scale defined by Ashcroft et al. (2) and modified by Hübner et al. (8). Three sections for each lung sample were stained with TM and scored on a scale of 0 to 8. The final score was expressed as a mean of individual scores observed across all microscopic fields. To quantify the distribution of pulmonary fibrosis, the Ashcroft scores were graded in three classes of increasing values ranging from 0 to 3 (mild), 4 (moderate), and ≥5 (severe).

To confirm the fibrosis assessment, the collagen content was measured using the image analysis software NIS-Elements AR 3.1 (Nikon Tokyo) on the TM-stained lung sections after selection of a correct green threshold detected on the Light Green stained collagen fibers to eliminate air spaces and bronchial epithelium.

After the whole slide analysis, due to the preferential localization in the subpleural portion of the lesions, we focused the fibrosis evaluation on the customized subpleural region of interest named Frame. The outer perimeter of the lungs was automatically detected following the edge of lung pleura, and afterward, a parallel to the previous line was allocated by NIS-AR image analysis software shifted/deepened 250 μm into the pulmonary parenchyma. The area of the lung contained between these two lines has been defined as Frame. The Ashcroft score, as previously described, was applied to the Frame area. Afterward, within the same area, fibrotic foci were pinpointed using a semiautomatic tool, which is morphologically and color-thresholding based; these portions have been defined as areas of interest (AOI). Two histomorphometrical parameters of AOI have been considered: the number and the AOI within Frame. AOI were summed for each sample and then normalized on the Frame surface (ΣAOIs/Frame). To evaluate the foci features, the areas of AOI were plotted in two classes, small (area ≤7,500 µm^2^) and large (area >7,500 µm^2^). For the skin samples, the histomorphometric parameters considered were dermis thickness, defined as the mean distance between the epidermal-dermal junction and the dermal-subcutaneous junction (13), and the hypodermis thickness, defined as the mean distance between the dermal-subcutaneous junction and the muscle layer. Measures were carried out at five randomly selected fields from one sample from each animal.

To detect M2 polarized macrophages in the lung tissue, paraffin-embedded sections were deparaffinized and rehydrated, and then antigen retrieval was performed in a 10 mM citrate buffer and kept at a boiling point. After a cooling step, the slides were rinsed in a wash buffer and then immersed in a blocking buffer (0.3 M glycine, 5% bovine serum albumin in 1× PBS; Sigma-Aldrich) at room temperature. Sections were incubated using anti-CD206 antibody (1 µg/mL, AF2535; R&D Systems), followed by incubation using Alexa Fluor 488 donkey anti-goat (3 µg/mL, AB_2336933; Jackson Laboratories). The nuclei were stained with DAPI. For negative control, the primary antibody was omitted, and tissues were incubated in 10 mM phosphate buffer or, alternatively, with unlabeled rabbit IgG nonimmune isotype control (2009-1; Alpha Diagnostic International) used at the same concentration of the selective antibody. Fluorescent WSI were acquired using NanoZoomer S60 (Hamamatsu Photonics, K.K.).

#### Data analysis.

Data are expressed as means ± SD. Statistical analysis was performed using one-way ANOVA followed by Dunnett’s *t* test, to compare each group versus the control group, or by Tukey’s, for multiple comparisons between groups. The comparison between the test and control groups during the different time points was by means of a repeated two-way ANOVA test and followed by a post hoc multiple comparison test for continuous data. The comparison of the frequency distribution was performed by a Chi-squared test. Statistics were carried out using Past statistical software package (version 3.0; https://folk.uio.no/ohammer/past) (5). A value of *P* < 0.05 was considered statistically significant.

## RESULTS

The first goal of the study was to identify the best dose of BLM to be administered to C57BL/6 mice.

Five mice were subcutaneously implanted with osmotic minipumps for each BLM dose (30, 45, 60, 75, and 90 U/kg). Mice were monitored and weighed daily. The effect of different BLM concentrations on lung histology, histomorphometric parameters, and bronchoalveolar lavage content was evaluated on *day 28* after pump implantation.

**Fig. 2.**
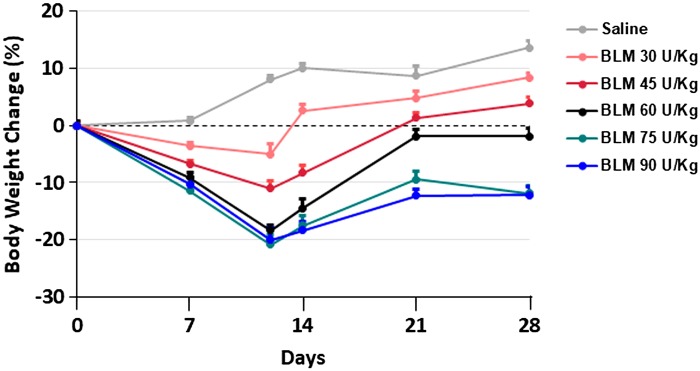
Effect of different dosages of bleomycin (BLM) on mouse body weight. Dose-dependent changes in body weight, on the indicated days, are expressed as a percentage of the weight measured on the first day of the experiment. Data points are means ± SD for 5 mice per group. All of the BLM doses caused a significant decrease in body weight compared with the saline group at all time points (two-way ANOVA test followed by Dunnett’s post hoc multiple comparison test for continuous data. *P* < 0.01).

In a dose-dependent manner, BLM induced a body weight reduction in the first 2 wk after pump implantation, with a recovery on *day 21* for the 30, 45, and 60 U/kg doses, and a slower recovery for the 75 and 90 U/kg doses (Fig. 2). No mortality has been observed.

The lungs of all BLM groups were histologically characterized by the thickening of alveolar septa, hypertrophic atypical epithelial cells, an increased number of alveolar macrophages, no marked inflammatory infiltrate, and, exclusively in the subpleural area, fibrosis coupled with narrowed alveolar spaces and re-epithelialization (Supplemental Fig. S2).

**Fig. 3.**
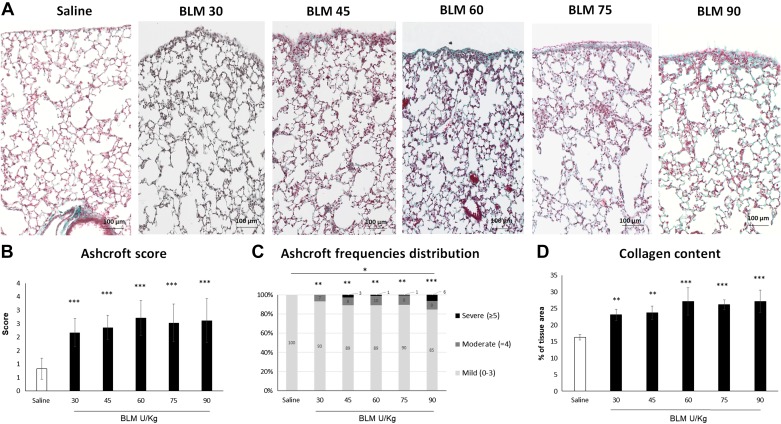
Effects of increasing dose of bleomycin (BLM) on lung fibrosis. *A*: representative microphotographs of the effects of saline and BLM at different doses (30, 45, 60, 75, and 90 U/kg) on lung parenchyma (×20 magnification). *B*: Ashcroft score determination. Data are shown as mean values ± SD for 5 mice per group. Statistical analysis was performed using ANOVA followed by Dunnett’s *t* test for comparison with the saline group and ANOVA followed by Tukey’s *t* test for comparison between different doses. *C*: frequency distribution of the Ashcroft scores values grouped as mild (0–3), moderate ( = 4) and severe (≥5). Statistical analysis was performed using Chi-squared test for independence. *D*: collagen content determination expressed as a percentage of the tissue area. Data and results of the statistical analysis are shown as in *B*. In each graph the asterisks above the bars indicate significant differences from saline and the asterisks above the horizontal lines indicate significant differences in the comparison between two different dosages (***P < 0.001; **P < 0.01; *P < 0.05).

**Fig. 4.**
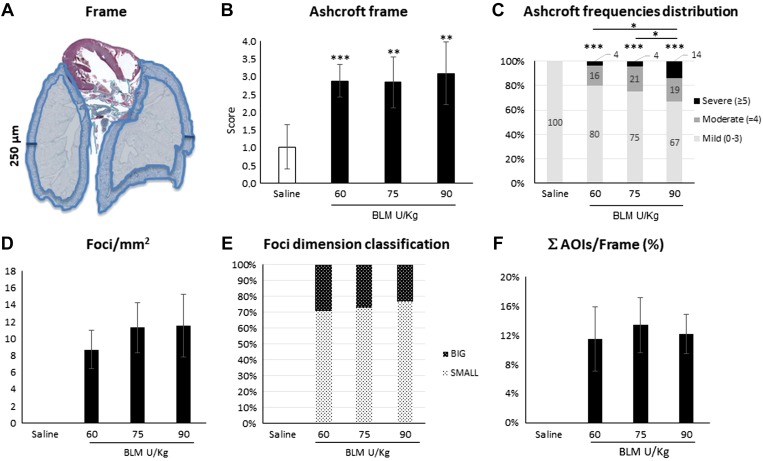
Histomorphometric analysis on the subpleural frame of mice treated with bleomycin (BLM; 60, 75, and 90 U/kg). *A*: schematic representation of the Frame 250-μm thick considered as a region of interest. *B*: Ashcroft score determination on the frame. Data are shown as means ± SD for 5 mice/group. *C*: frequency distribution of the Ashcroft score values grouped as mild (0–3), moderate ( = 4), and severe (≥5). *D*–*F*: foci metrics calculated as mean number of foci for mm^2^ of lung surface (*D*); foci dimension classified as small (area ≤7,500 µm^2^) and big area of interest (AOI) surface (area >7,500 µm^2^) (*E*), and AOIs normalized on the Frame surface (ΣAOIs/Frame) (*F*). Asterisks above the bars indicate significant differences in comparison to saline (ANOVA followed by Dunnett’s test, or Chi-squared test for comparisons between percentages). Asterisks above horizontal lines indicate significant difference in the comparisons between different dosages (ANOVA followed by Tukey’s test, or Chi-squared test for comparisons between percentages) (*P < 0.05; **P < 0.01; ***P < 0.001).

The fibrotic changes induced by the various BLM dosages exemplified in Fig. 3*A* were histologically evaluated via the Ashcroft score. The score was significantly higher for each BLM concentration in comparison to saline. No statistically significant differences were observed among the various doses (Fig. 3*B*). Frequency distribution of the Ashcroft score values grouped as mild (0–3), moderate (=4), and severe (≥5) revealed a prevalently mild fibrosis for each BLM dose. Comparing the effect of scaling dosages, the frequency distribution was significantly different from saline, but among BLM groups, the only statistically significant difference was found in the comparison between the lowest (30 U/kg) and highest (90 U/KG) doses (Fig. 3*C*). The assessment of fibrosis was confirmed by the results of the collagen content quantification (Fig. 3*D*). The tailored histomorphometrical analysis of the subpleural region named Frame was focused on the slides obtained from the groups treated with the highest BLM doses (i.e., 60, 75, and 90 U/kg), in which a more robust fibrotic response was achieved (Fig. 4*A*). The doses of 30 and 45 U/kg were instead discarded due to the lower Ashcroft score and collagen content.

The mean Ashcroft score values quantified in the Frame were increased compared with the previous analysis, but no statistically significant differences among the selected dosages were observed (Fig. 4*B*). Considering the frequency distribution, a significant increase of the highest degrees of fibrosis (namely, severe) was observed only in the group treated with the 90 U/kg BLM dosage (Fig. 4*C*).

In particular, when considering the key histological feature representing active fibroproliferation, the fibrotic foci, the comparison of their density, size and total areas did not highlight statistically significant differences among the different dosages, showing that the dose of 60 U/kg is already able to induce lesions comparable with those induced by higher doses (Fig. 4, *D* and *E*). Notably, the percentage of area occupied by the foci reported as ΣAOI/Frame (Fig. 4*F*) was found to be a more robust parameter of fibrosis than the same AOI surface normalized on the entire lung surface (data not shown).

The results of the BLM scale-up dose experiments, the histomorphometric analysis on the subpleural frame, and body weight recovery revealed that the 60 U/kg dose could be selected as the reference dosage for time course experiments, representing a fair compromise between lesions and animal welfare. Therefore, a total of 60 mice were implanted with osmotic minipumps delivering either saline or BLM at 60 U/kg, and five mice per group at selected time points (7, 14, 21, 28, 35, and 42 days) were euthanized (Fig. 1*B*). Differently from the previous experimental setup, besides the lungs, samples of skin, liver, and kidneys were also collected for histological analysis.

**Fig. 5.**
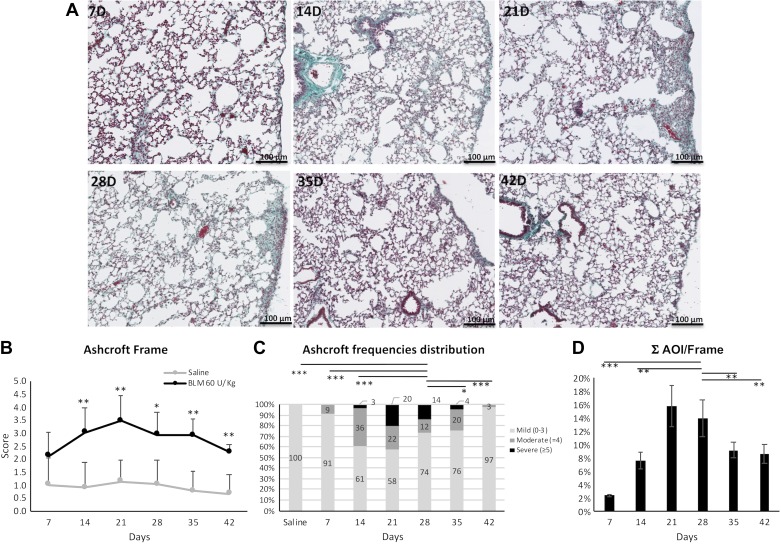
The time course of lung fibrosis in mice treated with bleomycin (60 U/kg). *A*: representative microphotographs of lung parenchyma at different time points. (×20 magnification). *B*: time course of the Ashcroft score. Data are shown as mean values ± SD for 5 mice per group. Changes were compared with vehicle groups using ANOVA followed by Dunnett’s test. *P < 0.05; **P < 0.01. *C*: frequency distribution of the Ashcroft score values grouped as mild (0–3), moderate ( = 4), and severe (≥5). Asterisks under horizontal lines indicate statistical significance (*P < 0.05; ***P < 0.001; Chi-squared test) in the comparisons between *day 28* and other time points or saline. *D*: time course of the surface occupied by fibrosis foci [ΣAOI (areas of interest)/Frame]. Changes were compared using ANOVA followed by Tukey’s test. Only the results of the comparisons between *day 28* and the other time points are shown. Asterisks under horizontal lines indicate statistical significance (**P < 0.01; ***P < 0.001).

Time course analysis (Fig. 5*A*) revealed that in the Frame region the fibrotic process had already started on *day 7*, when only isolated and gentle fibrotic changes, localized mainly on pleural and subpleural area, were detectable. On *day 14*, some portions of the subpleural region started to show fibrosis with the deposition of extracellular matrix and the thickening of the alveolar walls. On *day 21* the fibrotic lesions became confluent, albeit not larger in area, whereas many alveolar septa, scattered in the parenchyma, moderately thickened. Foci were organized mainly in longitudinal bundles parallel to the pulmonary surface, whose pleural width thickened and went deeper into the pulmonary parenchyma.

Between 21 and 28 days after the implant, the persistence of confluent fibrotic foci was visible. A slow, spontaneous resolution was observed at 35 and 42 days.

In accordance with histomorphometric observations, the Ashcroft score of the Frame changed considerably at each time point as compared with the saline-treated group, starting from *day 14* and peaking on *day 21* (Fig. 5*B*). Significantly higher percentages of severe Ashcroft scores (>5) were present only between *days 21* and *28*, whereas a marked reduction of severe scores were detected at later time points (Fig. 5*C*). The saline-treated group, as expected, showed normal lung architecture at all points of observation (data not shown).

The percentage of area occupied by the foci, reported as ΣAOI/Frame, was significantly higher in BLM-treated mice compared with the saline group. Indeed, similar to the Ashcroft trend, higher values of foci surface (AOI) were reached between *days 21* and *28* (>10%), with a subsequent reduction up to *day 42* (Fig. 5*D*). This confirmed that the analysis of the histomorphometric parameters of the Frame area could also be suitable for studying the evolution of pulmonary lesions during the time course.

**Fig. 6.**
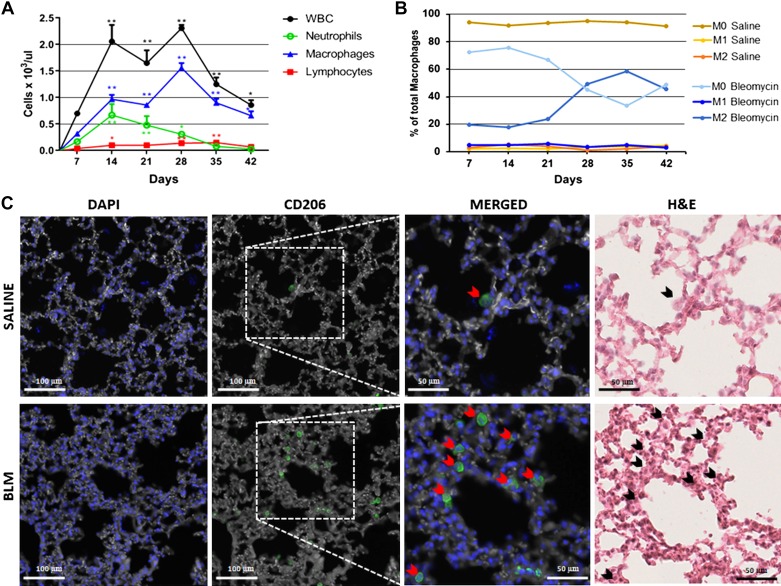
The time course of cellular infiltration in the lung of bleomycin (BLM; 60 U/kg)-treated mice. *A*: concentration of inflammatory cells in the bronchoalveolar lavage fluid. WBC, white blood cell. Data are shown as mean values ± SD for 5 mice per group. Asterisks indicate significant statistical differences in comparison with saline at each time point (*P < 0.05; **P < 0.01, ANOVA followed by Dunnett’s test). *B*: percent composition of macrophage polarization by FACS analysis. *C*: representative microphotographs of the lung tissue of saline and BLM-treated mice on *day 28* after immunofluorescent staining for DAPI and CD206 (×20 magnification). The merged images and those stained with H&E are enlargements (×40 magnification) of the area bounded by dashed rectangles. The red arrowheads indicate CD206+ cells in immunofluorescence images. The same cells in the H&E images are indicated with black arrowheads.

Pulmonary inflammation was evaluated by counting, at each time point, the number of inflammatory cells in the BALF of mice either implanted with osmotic minipumps delivering saline or BLM (60 U/kg). The white blood cell (WBC) number, on *days 14*, *21*, *28*, *35*, and *42*, was much higher in BLM- than in the saline-treated groups (Fig. 6*A*). WBC numbers peaked on *day 14* and remained quite stable up to *day 28*. Neutrophils fraction was characterized by a significant increase on *day 14*, followed by a moderate decrease in favor of the macrophage fraction. Lymphocytes at each time of treatment remained at basal level (Fig. 6*A*).

To further characterize individual cell population, we used a flow cytometry technique using specific surface markers. Our goal was to verify the polarization of macrophages, the predominant population that infiltrated the lungs. The remaining BALF from the cell count was used for FACS staining. Lymphocytes and neutrophils were identified based on their FSC and SSC properties and as F4/80− cells. A population gated as F4/80+CD11b+ cells, likely representing macrophages, increased at each time point in mice delivered with BLM compared with saline-treated control.

To study macrophage polarization toward the M1 or M2 subpopulation, the percentage of cells expressing MHCII or CD206 was evaluated in gated cells F4/80+CD11b+. In the BALF of the saline group the predominant macrophage population was unpolarized (M0), detected as negative for MHCII and CD206 markers. In the group treated with BLM, the increased CD206 expression within the macrophage F4/80+CD11b+ population indicated the presence of M2-like polarized cells, particularly at 28 days, whereas the M1-like (MHCII+ CD206- cells) population showed low numbers at all time points (Fig. 6*B*). An increased number of CD206 immunofluorescent M2-like macrophages was also observed in lung tissues of BLM-treated mice compared with control mice (Fig. 6*C*). The expression of cytokines, chemokines, MMPs and TIMP-1 was monitored during the 3 wk of higher inflammatory response. Changes in the expression levels of IL-4, IL-10, IL-12, IL-13, and G-CSF were observed in the BLM-treated mice compared with control mice (Supplemental Fig. S3*A*). No significant variation was observed in the levels of any other cytokines or chemokines tested (data not shown). In particular, the cytokine levels relevant for M2 polarization such as IL-13 and IL-4 (Th2 cytokines) significantly increased on *days 14* and *21*, respectively, compared with saline (green dashed line in Supplemental Fig. S3), suggesting their role in driving the macrophage switch toward the M2 phenotype in the group treated with BLM. Moreover, at each of the three experimental stages (14, 21, and 28 days) a significant rise was observed compared with the control mice only for G-CSF, whose mean value, however, appeared to slightly decrease during the course of time, and for IL-10 and Il-12, which peaked on *day 28*, concurrently with the macrophage peak (Supplemental Fig. S3*A*). With regard to the BALF levels of the MMPs and TIMP-1, these were in general significantly higher in BLM-treated mice. MMP2 was characterized by a constantly high level during the time course, whereas MMP-9 had a decrease on *day 21*, followed by a moderate increase on *day 28*, which was associated with a reduction in TIMP-1 levels (Supplemental Fig. S3*B*).

**Fig. 7.**
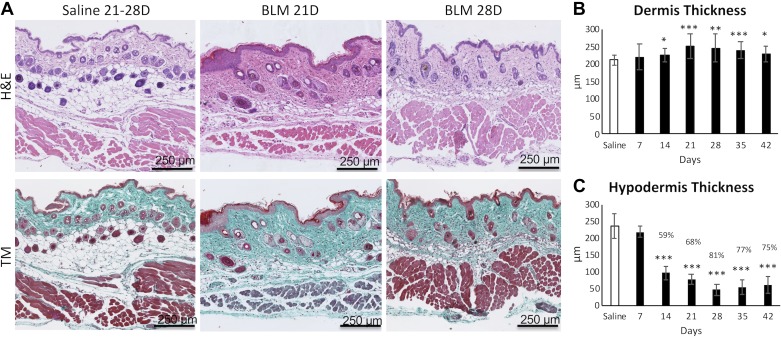
Histomorphometric analysis of dermal fibrosis. *A*: representative microphotographs of dermal fibrosis and reduction of the cell adipose layer induced by bleomycin (BLM; 60 U/kg). H&E (*top*) and Masson trichrome (TM; *bottom*) staining of sequential skin sections (original magnification, ×10). *B* and *C*: dermis thickness (*B*), expressed as the mean distance between the epidermal-dermal junction and dermal-subcutaneous junction, and hypodermis thickness (*C*), expressed as the mean distance between the dermal-subcutaneous junction and muscle layer, were measured at each time point in the skin of the gluteal region of 5 mice per group. From *day 14*, mice receiving the continuous BLM infusion with minipumps exhibited significantly increased dermal thickness and reduced adipose layer thickness compared with saline-treated controls. Asterisks indicate statistical significance (*P > 0.05; **P < 0.01; ***P < 0.001; ANOVA followed by Dunnett’s test). Reductions of the adipose layer thickness greater than 30%, compared with saline, are indicated in the histogram.

The skin histology of BLM-treated animals showed a fibrosis-like pattern characterized mainly by increasing collagen compactness within the dermis connective tissue, with fibers starting to penetrate the space previously occupied by adipocytes due to the severe lipoatrophy of the underlying adipose layer. These lesions were concurrently detected during the time course from *day 14* up to *day 42* (Fig. 7*A*). Moreover, hypertrophy of epidermis and in hair follicles was also observed (Supplemental Fig. S4). Histomorphometry showed a significant increase induced by BLM in dermal thickness compared with the saline group (Fig. 7*B*), with an almost complete loss of the adipose cell layer and a simultaneous decrease of hypodermal thickness (Fig. 7*C*).

Regarding other target organs such as liver and kidneys, no morphological changes were observed with the 60 U/kg BLM dosage in the connective tissue of either stromal or parenchymal components compared with the saline treated groups (Supplemental Fig. S5).

## DISCUSSION

The present study describes a murine model usually used for SSc and related ILD, in which BLM is administrated through osmotic minipumps implanted subcutaneously. Very few studies had already proposed a similar approach (6, 12, 13, 28) but assessed lung fibrosis using conventional histological methods, which are not able to discriminate slight pulmonary structural changes caused by different BLM concentrations or which arise during time. To learn more about the development of pulmonary fibrotic-like changes, we based our model on C57BL/6 mice that, although not the strain of choice for dermal fibrosis studies, are more susceptible to the induction of lung fibrosis compared with other strains (6, 28). A scale-up of the BLM dose was performed, and, out of the five different concentrations tested, 60 U/kg was selected as the best dose to study the time course of fibrosis progression over a period of 42 days. The selected dose induced a sufficient and reproducible fibrosis in the skin and lung but was probably too low to cause detectable morphological changes in the kidney and liver. Higher concentrations of BLM did not induce a further increase in lung fibrosis but had a significant impact on the animal’s welfare and body weight.

This was not a foregone result, since two studies on the BLM pump model using C57BL/6 mice reported contradictory data: “no weight loss or mortality” was observed delivering a 225 U/kg BLM dose (sex of mice not declared) (13), by contrast, Harrison and Lazo (6) highlighted high mortality rate (25%) delivering a 150 U/kg BLM dose to female mice (6). Regarding the sex, the American Thoracic Society suggests the use of male mice, since they are more susceptible to BLM treatments and should require lower BLM concentrations compared with females (9). However, the National Institutes of Health recommends use of both male and female animals in all research studies (9, 22). In our study, we used female mice because they are much less aggressive than males and do not require frequent interventions to separate the dominant.

In our pump model, at 60 U/kg of BLM, lung fibrosis appeared mild and restricted to subpleural areas, with histological features similar to the ones described by Harrison and Lazo (6); however, the lung disease persistence was shorter in time compared with their study (6) and not sustained by chronic inflammation.

Furthermore, we registered the peak of weight loss within 14 days from the beginning of BLM administration and then a gradual recovery of body weight in the following days.

In the study by Harrison and Lazo (6), the only one that investigated experimental times longer than 4 wk and used female mice, despite cessation of BLM infusion after the first week, progressive loss of lung function, collagen accumulation, extension, and confluence of lesions in a large fraction of the subpleural area were observed between *days 28* and *42* posttreatment. Moreover, mice died between *days 37* and *41* posttreatment after developing severe pulmonary fibrosis. Because the strain, sex, and age of mice were the same as in our study, the differences observed could be due to the lower dose of BLM that we used.

Considering the mildness of the induced fibrosis and the subpleural pattern of foci distribution, the classical histological approach, which requires subjective choices of microscopic fields to analyze and locations to take measurements (6, 12, 13), has been refined with the aim of better detecting and quantifying the fibrotic foci in the lung parenchyma. The whole slide imaging coupled with semiautomated image analysis, used as a new tailored analysis of the subpleural areas, enabled us to detect and quantify small changes in the lung parenchyma caused by different BLM doses, and therefore, it appeared to be a reliable histomorphometric method, potentially even applicable to drug screening.

Applying this analysis to the time course experiment, it was possible to gain insight into the evolution of fibrosis progression and identify its peak between *days 21* and *28* after treatment with the dose of 60 U/kg. So, despite a trend toward spontaneous resolution, it was possible to recognize a therapeutic window for testing pharmacological compounds. Within this time frame the fibrotic lesions occurred almost exclusively in the subpleural area. The pattern of fibrosis was characterized mainly by confluence of fibrotic foci infiltrated by M2 macrophages, detected by immunofluorescent staining, and only limited inflammation. This was in accord with the literature about this model system (12). BALF analysis in the time course study revealed an initial increase in the WBC numbers followed by a decrease, whereas the fibrosis development occurred as a secondary effect. The WBC differential composition analysis showed only a transient increase of neutrophils and a more marked and sustained increase of monocytes. This fraction was composed mainly, up to *day 21*, by uncommitted macrophages, which on *day 28* differentiated mainly toward the M2 phenotype, which influences angiogenesis and tissue remodeling and repair and produces profibrotic mediators (18). As expected, their increase in BALF of BLM-treated mice was preceded by an increase in IL-4 and IL-13 levels (16) and coincided with the peak of the anti-inflammatory cytokine IL-10 that was involved in the alveolar macrophage phenotype shift to M2 (21).

Another histological feature of the model was the presence of hypertrophic type 2 pneumocytes, which would also relate to the lung fibrosis (12). According to Adamson and Bowden (1), in fact, the antineoplastic drug would first cause in the lung a selective injury of rapidly dividing epithelial cells, like endothelial cells and type 1 pneumocytes. Type 2 pneumocytes, more resistant to the drug thanks to their slower turnover (it takes ∼3 days), would activate themselves to repair the air-blood barrier; however, if the toxic agent is still present at the time of their division, as it is in continuous infusion through osmotic pumps, they would become susceptible to injury. When damage occurs, these cells begin to secrete chemokines and cytokines, which in turn activate resident fibroblasts, enhancing the fibrocyte migration and epithelial-mesenchymal-transformation (10) and eventually promote fibrosis without inciting inflammation.

In accord with clinical studies reporting elevated levels of MMP and their tissue inhibitors in IPF patients (4), the BALF analysis showed constant overexpression of MMP-2 and MMP-9 in comparison with control animals, with the exception of a transient decrease of MMP-9 on *day 21*. This variation, corresponding to the peaks of WBC in the BALF cell count, can be explained with the fact that MMP-9 was expressed mainly by inflammatory cells, whereas MMP-2 was expressed by epithelial cells (20). As opposed to other interstitial collagenases that spread proteolysis, MMP-2 and MMP-9 are localized on the cell surface and cleave denatured collagens (gelatinases) and basement membrane proteins (4). Therefore, they also could promote abnormal epithelial repair processes in fibrotic lungs of BLM-treated mice. Their tissue inhibitor, TIMP-1, which was expressed in the interstitial cells of fibrotic areas during the reparative phase (20), instead maintained a high level in the BALF up to *day 21* alongside the presence of the peak of activated macrophages in the alveolar spaces but decreased at 28 days, probably following a progressive depletion of cellular reserves of the inhibitor (20).

Finally, our model allows us to observe the induction of dermal fibrosis, even in the early phase of treatment, and its progression and persistence up to *day 42* in C57BL/6J mice. Other studies using the same mouse strain and BLM dose reported only a transient dermal fibrosis near the implantation site within the first 10 days, with no variation in the abdominal skin (28). Higher doses instead produced both inflammatory infiltrates and a significant increase in dermal thickness, starting in the first week and peaking on *day 28*, in the skin just above the pumps (13). According to Watanabe et al. (28), these differences could depend both on the BLM dose and on the distance from the implantation site. The increased dermal thickness was characterized by high compaction and parallelism of collagen fibers, inflammatory cell infiltration, and a wide lipoatrophy. This last phenomenon became significant starting from the second week from the beginning of BLM delivery and is a peculiar feature occurring also in scleroderma patients. Our observations are consistent with recent findings (14), according to which, in mouse models of dermal fibrosis induced by BLM, there would be, first of all, a loss of dermal white adipose tissue (DWAT). In most areas of mouse skin, DWAT is located between reticular dermis and panniculus carnosus, whereas in other parts, like in humans, the panniculus carnosus is not present; therefore, DWAT, hair follicles, and sebaceous glands can partially infiltrate the subcutaneous white adipose tissue. DWAT contains adipocytes that can transdifferentiate to myofibroblasts contributing to the formation of fibrotic tissue and then, during resolution of wound healing, can newly transdifferentiate into adipocytes.

In conclusion, we have set up a murine BLM pump model inducing lung and skin fibrosis that seems very promising for testing putative antifibrotic drugs under a therapeutic regime. The limited weight loss and lack of mortality observed should be considered as a great advantage allowing for better planned experiments with a reduced number of mice. The development of pulmonary fibrosis is progressive, even if only for a limited time, and is not sustained by chronic inflammation. In this perspective, our model resembles IPF, a disease in which inflammation is not a major driver of its progression. Fibrosis is characteristically mild and limited to the pleural and subpleural portion of the lung, making the model more similar to human scleroderma associated ILD. The choice of evaluating the whole peripheral part of the lung, instead of a limited number of frames, with a semi-automated tool allows a better overview, a more objective evaluation and heightened sensitivity, in comparison to the histological analysis applied to other pump models. Despite the tendency to spontaneous disease resolution, the fibrosis process was already established in both lung and skin at *day 14* and remained stable until *day 35*. Therefore, one of the most challenging aspects is the window of about three weeks for drug intervention where compounds could be tested, as single agents or in combination, to mimic the clinical situation.

## GRANTS

This work was supported by internal funds from Chiesi Farmaceutici S.p.A.

## DISCLOSURES

No conflicts of interest, financial or otherwise, are declared by the authors.

## AUTHOR CONTRIBUTIONS

F. Ravanetti, L.R., R.C., F. Ruscitti, and F.F.S. conceived and designed research; F. Ravanetti, L.R., R.C., F. Ruscitti, D.P., and F.F.S. performed experiments; F. Ravanetti, L.R., R.C., F. Ruscitti, and F.F.S. analyzed data; F. Ravanetti, L.R., R.C., F. Ruscitti, and F.F.S. interpreted results of experiments; F. Ravanetti, L.R., R.C., and F. Ruscitti prepared figures; F. Ravanetti, L.R., F. Ruscitti, and F.F.S. drafted manuscript; F. Ravanetti, L.R., R.C., F. Ruscitti, G.V., and F.F.S. edited and revised manuscript; F. Ravanetti, L.R., R.C., F. Ruscitti, F.G., G.V., A.C., and F.F.S. approved final version of manuscript.
